# Perspectives on the Efficacy of Antidepressants for Child and Adolescent Depression

**DOI:** 10.1371/journal.pmed.0030009

**Published:** 2005-12-20

**Authors:** Amir Raz

## Abstract

Raz looks at the ongoing controversies surrounding the use of SSRI antidepressants in children.

Practitioners of pediatric medicine may still be undecided as to whether the newer generation of antidepressant drugs is effective for child and adolescent depression (CAD) [1]. Since 1989, when selective serotonin reuptake inhibitors (SSRIs) were introduced in the United States, they have become the top-selling drug category; as many as one in eight adult Americans having tried at least one SSRI in the past ten years. Despite their popularity in treating adult depression, the efficacy of SSRIs for CAD remains in dispute. In this article, I examine some of the core problems in medical research that have led to this disagreement.

## Specificity, Safety, and Efficacy

Advances in molecular biology and neuroscience have fostered increasingly specific drugs. However, the pharmaceutical industry promotes an idea of drug specificity that may extend beyond the existing data. For example, SSRIs may selectively block the reuptake of serotonin, as claimed by many SSRI manufacturers, but they also influence numerous postsynaptic serotonin receptor systems, instigating multiple neurochemical effects. Furthermore, certain neurotransmitter systems are so tightly entwined that affecting one inevitably influences others (e.g., selective norepinepherine reuptake inhibitors also influence the serotonergic system). Hence, drugs often have effects that seem unrelated to the presumed therapeutic outcome (e.g., tricyclic antidepressants [TCAs] and SSRIs have significant effects on fast sodium channels and platelet function, respectively). And one drug can treat a variety of syndromes. For example, SSRIs are effective for symptoms ranging from obsessive-compulsive disorder to panic and anxiety. Thus, specificity, as defined by the pharmaceutical industry, is perhaps an overextended notion.

Antidepressant medications have become central to managing CAD [2]. Because double-blind trials of TCAs have failed to show greater efficacy than placebo for treating CAD [3,4], and concerns have been raised about the side effects of TCAs, SSRIs have been seen as the viable option for treating CAD [5]. Indeed, the 21st century ushered in major clinical guidelines endorsing SSRIs as first-line pharmacotherapy for CAD in both North America and the United Kingdom [6,7]. Most rigorous studies that tested the safety and efficacy of these medications in depressed adolescents began after these drugs were deemed “first line” by the professional community of child and adolescent psychiatrists.

Yet the recent history of SSRIs is replete with inconsistent verdicts about their safety. For example, in January 2003, the US Food and Drug Administration (FDA) approved fluoxetine for children and adolescents. However, about five months later, concerns arose among psychiatrists about whether the drug was associated with suicidal thinking and behavior in children and adolescents. Nevertheless, in December 2003, the UK Medicines and Healthcare Products Regulatory Agency (MHRA) supported the use of fluoxetine in children and adolescents [8,9]. It stated that for three other SSRIs (sertraline, citalopram, and escitalopram) the risks outweighed the benefits, while the balance of risks and benefits was “unassessable” for a fourth SSRI, fluvoxamine [8]. In September 2004, based on a review of 24 trials of nine different antidepressant drugs that were used to treat CAD, obsessive compulsive disorder, or “other psychiatric disorders,” the FDA also supported the use of fluoxetine in treating CAD [10]. Prior to this, on March 22, 2004, the FDA had issued a “black box” warning label on all antidepressants, cautioning that these medications may “increase the risk of suicidal thinking and behavior (suicidality) in children and adolescents with major depressive disorder (MDD) and other psychiatric disorders” [11].

In its September 2004 review, the FDA endorsed SSRI safety as well as an arbitrary improvement criterion—some decrease, relative to placebo, on a reputable scale (e.g., a two-point drop on the Hamilton depression scale)—without addressing actual clinical efficacy. However, the resulting assumption among some practitioners was that SSRIs in general are effective, as well as safe, for CAD. This conclusion is consistent with recent reviews [12,13], drawing on both published and unpublished data, reporting that at least fluoxetine is seen as a safe and efficacious treatment for CAD. The tendency to embrace SSRIs for CAD demonstrates a trend in pediatric mental health practice toward taking efficacy for granted and focusing on safety.

## Statistical Significance versus Clinical Significance

Paying too much attention to significance tests and too little attention to the analysis methods [14] or other aspects of the data (e.g., the estimates of the magnitude of the effects [15]) may blur the difference between statistical significance and clinical importance [16,17]. Furthermore, statistical significance itself can be clinically meaningless.

For example, a hypothetical study with a large sample size might show that an average heart rate of 69 on placebo compared to 71 on a drug is statistically significant, but this effect is likely to be clinically meaningless. In fact, the only standard for determining drug efficacy for “quality-of-life illnesses,” including CAD, is a placebo-controlled trial; a comparator (i.e., a “horse-race,” or “drug A versus drug B”) trial typically boosts the drug effect (P. Roose, personal communication and [18]). (This is partly due to the fact that when people know that they are being treated with either a more or less potent medicine, drug response tends to be more vigorous than when they know that they may be on either an actual drug or a placebo). Finally, scientific experiments rarely control for variables known to influence drug response (e.g., expectation, suggestion, motivation, site location, and trial length) (P. Roose, unpublished data).

Clinical significance—a meaningful change in the symptomatic state or functioning of an individual patient—requires independent replication of results [19]. Different fields have different criteria for clinical significance. Additionally, the statistical method used to calculate clinical significance affects the estimates of meaningful change [14]. Indeed, some professional associations (e.g., the American Psychological Association) have deemed effect sizes and confidence intervals to be more meaningful measures than significance testing [20].

To determine clinical significance through “risk-benefit” analysis [21], one must weigh the potential benefit of improved symptomatology, accompanied by adverse side effects, against the risk of leaving the disease untreated. Trying to address the issue of clinical significance, researchers have considered such parameters as the number needed to treat (NNT), the number needed to harm, and the number needed to prevent [22]. In the case of depression and CAD, FDA approval of fluoxetine implies that the FDA considered the number needed to harm to be reasonable. But what is a good value for NNT? Ideally, it would be as close as possible to one (i.e., we need to treat only one person in order to see a desired effect in one person), but actually the NNT tends to be much higher than one [23], and it is unclear what range of values permits clinicians to conclude that a favorable benefit-to-risk ratio exists.

In addition, the NNT must be interpreted by using a comparison group. For example, in a placebo controlled trial, NNT = 3 means that on average one of three patients will derive specific benefit from the treatment above and beyond placebo, which is rarely used clinically. Thus, NNT may be more clinically meaningful as an active comparator than in relation to a placebo. The absence of clear criteria for clinical efficacy is probably partially responsible for interpretation of the same data as being both for [24] and against [25] the efficacy of a specific drug.

Lacking clear criteria for clinical significance, statistical significance is perhaps the most convenient substitute [26]. But researchers may pay too much attention to the results of significance tests, thereby overlooking clinical significance. Statistical significance may not be a sufficient criterion for recommending a drug [27]. In the case of adult antidepressants, FDA approval requires that two randomized clinical trials (RCTs) show that drug performance is at least two Hamilton-scale points better than a placebo; however, this arbitrary criterion does not signify clinical efficacy. In fact, it is unclear what criteria should be used to assess clinical significance. For example, how many studies are needed to convince a clinician that a drug is efficacious? Clinical significance relies on replication and probably requires a meta-analysis. Recent [21,50] as well as future meta-analyses, including one currently under preparation by A. Drews, I. Kirsch, and D.O. Antonuccio entitled “A Meta-Analysis of Antidepressants Trials for Depressed Children: Small Benefits, Large Stakes”, may further illuminate the efficacy of antidepressants for CAD.

## Belief Systems

While strong opinions on either side of any controversy may appear extreme, it is important not to disregard these beliefs immediately. For example, on February 2, 2004, Irving Kirsch and David Antonuccio offered their testimony to the FDA on the efficacy of antidepressants for treating children with depression. At that time, only a dozen RCTs had examined the efficacy of antidepressants in CAD (four assessed SSRIs, seven assessed TCAs, and one assessed both SSRIs and TCAs) [28–38] (see sidebar). Eight of these RCTs failed to find any significant benefit of medication over placebo. While no TCA-placebo comparisons showed significant differences, four of the five SSRI-placebo RCTs (plus a fifth that included SSRIs and TCAs) claimed significant differences between drug and placebo, but only on clinician-rated, not patient-rated, measures.

**Incentives for Conducting Pediatric Clinical Trials**

Giving pharmaceutical companies an incentive to conduct pediatric tests, Congress passed the FDA Modernization Act in November 1997. Section 111 of this “pediatric exclusivity” partnership act offered drug sponsors six months (sometimes up to a year) of additional market exclusivity if they conducted pediatric studies on drugs still under some exclusivity provision. Under the FDA Modernization Act, the pharmaceutical company could continue to set the market price, keeping generic forms of the drug off the market. The original act has since been revised and extended through 2007 under the Best Pharmaceuticals for Children Act, but concerns still linger regarding disproportionate profits accrued by drug companies from the six-month extension compared to the cost of clinical trials, and the absence of commitment by the companies to publish or make readily available the safety and efficacy results of these trials [92]. While it is easy to see why pharmaceutical companies are eager to conduct pediatric research (six-month worldwide exclusivity for fluoxetine, for example, is estimated to be worth about a billion dollars), clinicians and patients rarely have access to these findings [93].

Since either means or standard deviations were missing in 25% of these RCTs, only nine were amenable to meta-analytic scrutiny. When Kirsch and Antonuccio combined data from these nine studies for analysis, the placebo response was 87% of the drug response, 75% of the SSRI response, and 97% of the TCA response. These results seem to indicate that TCAs have no significant pharmacological effect on CAD. They concluded that the effect of SSRIs may be statistically significant, but possibly not clinically significant.

While some psychopharmacologists dismiss investigators such as Kirsch and Antonuccio as “outliers” or inherently biased against the drug industry, such “outlying” accounts should nevertheless be examined. Opinions on either side of this issue should be considered, especially in the absence of definitive data and facts.

## Market Forces

In the US, the pharmaceutical industry, Congress, and advocacy groups are known to both lobby and contribute generously to the FDA and may partially inspire its decisions and policies (P. Roose, personal communication). The influence of the pharmaceutical industry permeates science [39], and evidence points to the increasing commercial impact of biomedical research on scientific reporting [40,41]. Indeed, industry funding for research tends to yield favorable reports concerning the tested drug [42]. For example, among the authors of original research papers, reviews, and letters to the editor that were supportive of the use of specific drugs, 96% had financial relationships with the drugs' manufacturers, whereas for publications deemed neutral or critical, the figures were only 60% and 37%, respectively [43,44].

Further, since negative results are often discounted or not published [45,46,47], the message conveyed to the popular press and the public is often positively skewed [48], emphasizing benefits over risks and predicting improbable breakthroughs [49]. This trend may create unrealistic expectations about scientific advances or products and may lead to inappropriate and expensive utilization patterns. However, commercial pressure is not the only source of ambitious interpretations; another is researchers who are eager to promote their latest findings. Thus, conflicts of interest in SSRI trials for treating CAD may potentially cloud results.

## Different Reviews Have Had Different Results

Although most previous reviews have been partisan, a few have presented a balanced account [1,50,51]. The first, though least comprehensive, review of RCTs conducted on newer antidepressants for CAD examined six published studies, including studies on venlafaxine and three SSRIs: fluoxetine (three studies), sertraline (one study), and paroxetine (one study) [52]. In their limited meta-analysis, the authors used a random effects model to pool averaged, selected outcomes across the five SSRI studies. They found a small effect size of 0.26 (95% confidence interval [CI], 0.13–0.40), which they described as equivalent to a three-to-four-point improvement on the revised children's depression rating scale (which ranges from 17 to 113). They concluded that a large benefit from newer antidepressant drugs is unlikely. Reviewing the same studies, another report judged the efficacy data to be inconclusive [53].

Since the above reviews [52,53] included in their analysis a negative study of fluoxetine which involved a small (n = 30), clinically heterogeneous (mixed inpatients and outpatients) participant group [39], some later reviews opted to exclude this negative study from their analyses. For example, one study analyzed five of the six published papers addressed in the above-mentioned reviews [52,53], as well as data from six unpublished studies accessed through collaboration with the UK's MHRA [50]. These unpublished studies included two investigations of venlafaxine, paroxetine, and citalopram, respectively (one of the citalopram studies was subsequently published [54]). After extracting raw data for outcome measures, including remission, response to treatment, and depressive symptoms scores, the authors reanalyzed, and—where possible—meta-analyzed published and unpublished studies of each drug, using fixed-effects and random-effects models. The authors concluded that only fluoxetine had evidence of efficacy that was robust: across two published trials, fluoxetine was more likely than placebo to bring about remission (number needed to benefit 6; 95% CI, 4–15), or a clinically meaningful response (number needed to benefit 5; 95% CI, 4–13). The study did not draw on unpublished fluoxetine efficacy data.

The most recent systematic review of newer antidepressants for CAD to date [55] included all of the data previously reviewed [50,52,53] as well as unpublished data regarding two RCTs of nefazodone, and the recently published Treatment for Adolescents with Depression Study (TADS) [23], which compared fluoxetine, placebo, and cognitive behavioral therapy (CBT) alone and in combination (see Table 1). After examining efficacy outcomes and the influence of methodology (site selection, study population, study design, and outcome measures) on those outcomes, the authors found that the more methodologically sound SSRI studies tended to have better outcomes. For example, in the TADS, 60.6% (95% CI, 51%–70%) of adolescents responded to fluoxetine alone, as opposed to a 34.8 (95% CI, 26%– 44%) response rate for placebo. Based on these findings and no evidence of differences among these drugs in adult populations, the authors concluded that most newer antidepressants are likely effective, and that different results have been largely due to methodological differences in studies of CAD. They concluded, therefore, that at least fluoxetine is clinically effective for CAD.

**Table 1:**
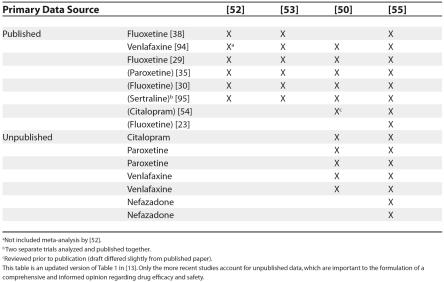
A List of Published and Unpublished Sources Commonly Cited in Recent Reviews Reporting on SSRIs for Child and Adolescent Depression

^a^Not included meta-analysis by [52].

^b^ Two separate trials analyzed and published together.

^c^Reviewed prior to publication (draft differed slightly from published paper).

This table is an updated version of Table 1 in [13]. Only the more recent studies account for unpublished data, which are important to the formulation of a comprehensive and informed opinion regarding drug efficacy and safety.

## Methodologic Oversights in Published Studies

The TADS concluded that “medical management of MDD with fluoxetine, including careful monitoring for adverse events, should be made widely available” [23]. However, some researchers disagree with this conclusion [25].

The TADS explores four experimental arms: placebo, fluoxetine, CBT, and CBT + fluoxetine. Some of these treatment groups were “blind” (i.e., participants were unaware of what treatment they were receiving, as in the case for placebo) while others were informed (as in the case for CBT). However, Table 2 shows the difficulty of interpreting a study that compares blinded and unblinded treatment groups (e.g., differences between treatment groups might be due to the varying influence of expectation). Thus, in the interpretation of the TADS findings, subtle methodological caveats go unrecognized.

**Table 2:**
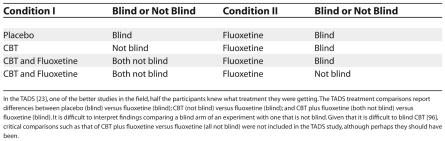
A Subtle Flaw in the TADS: Condition I versus Condition II

In the TADS [23], one of the better studies in the field, half the participants knew what treatment they were getting. The TADS treatment comparisons report differences between placebo (blind) versus fluoxetine (blind); CBT (not blind) versus fluoxetine (blind); and CBT plus fluoxetine (both not blind) versus fluoxetine (blind). It is difficult to interpret findings comparing a blind arm of an experiment with one that is not blind. Given that it is difficult to blind CBT [96], critical comparisons such as that of CBT plus fluoxetine versus fluoxetine (all not blind) were not included in the TADS study, although perhaps they should have been.

Relative to other studies, CBT does not fare well compared to both placebo and fluoxetine in the TADS. However, other studies comparing antidepressants and CBT showed that both were moderately effective in relieving depression in adults [56]. Adult neuroimaging findings suggest that the two methods work by improving the functioning of different brain circuits: CBT operates on cortical areas related to attention and comprehension, including the anterior cingulate cortex, whereas antidepressants operate on subcortical areas. Applying the adult data to children, these exploratory imaging results may provide both a context for testing drugs against nonpharmacological therapy and a basis for considering how to treat or even prevent depression in those who are most susceptible [57].

Despite being regarded as efficacious in adults, meta-analysis of published RCTs indicates that 75% of antidepressant response in adults is duplicated by placebo [58]. This initial meta-analysis was amply critiqued by Klein for multiple limitations [59]. However, follow-up analyses using a different data set taken from the FDA, to which Klein's objections do not apply, again reported that about 80% of the response to antidepressants in adults was duplicated in placebo control groups [60,61]. Together with the notion that antidepressant medication effects are typically weaker in children than adults [58,60], these conclusions accord with earlier reviews that challenge the effects of antidepressants in CAD [4,62–67]. Merely labeling a pill an antidepressant does not make it so. In fact, the existing data suggest that antidepressants are probably more effective in treating anxiety than depression [68].

Another limitation draws on the implications of using antidepressants in early life [69]. Serotonin acts as a brain and glial growth factor in early development. Some serotonin receptors act during development to establish normal anxiety-like behavior later in life, while others play a role in synapse formation [70,71]. Some exploratory findings suggest that artificial perturbation of serotonin function in early life may alter the normal development of brain systems related to stress, motor development, and motor control [71,72]. Furthermore, early exposure to fluoxetine produced abnormal emotional behaviors in adult mice [69]. The critical role of serotonin in the maturation of brain systems that modulate emotional function in the adult suggests that, in concert with genetic makeup, low serotonin levels during early development may increase vulnerability to psychiatric disorders [69,73]. Early exposure to SSRIs, therefore, can potentially exact a heavy price in later life [74,75].

These caveats suggest that, in addition to the potential implications of using antidepressants in early life, there are few compelling data sets, free of funding from drug companies, concerning the efficacy of antidepressant medications over and above their placebo value for CAD. These caveats also show the difficulty of assessing the clinical significance of the unique effects attributable to antidepressant medications. Because antidepressants work just slightly better than placebos, even according to data endorsed by the pharmaceutical industry, the image of antidepressants as more effective may be overreaching and perhaps a consequence of methodological artifacts [76].

## Public Health versus Individual Decisions

The US Surgeon General makes decisions based on the greater good of a vast population: a mere two-point improvement on the Hamilton depression scale may constitute a meaningful public-health benefit (D. Shaffer, unpublished data). However, parents decide whether their depressed adolescent child should receive CBT, fluoxetine, or start a vigorous exercise regime, based on an individually tailored risk-benefit analysis.

Most clinicians recommend psychotherapy for mild to moderate CAD and reserve SSRIs for severe CAD or when therapy is not effective [21]. Yet given the large numbers of people suffering from depressive disorders (i.e., an estimated 1.5 million adolescents (12–18 years of age) with MDD in the US alone [77]), it is easy to see why offering therapy would be difficult (e.g., number of therapists and insurance considerations), thereby making the drug option more popular.

Despite early suggestions in the literature [78–80], accounts of the association between adult suicidality and the use of SSRIs have been inconclusive [81–83]. One early meta-analysis showed that SSRIs potentially decreased suicidal ideation as measured by a single question on the Hamilton depression score [83], but a more recent study reported a non-significant increase in suicide rates between patients assigned to SSRIs and those assigned to placebo or other antidepressants [82]. Despite the FDA's black-box label, some accounts suggest that since the introduction of SSRIs the number of successful suicides has steadily declined [84,85]. One recent meta-analysis reported that SSRIs increased the risk of suicide attempts, but not completions, across all indications [86]. Another, a meta-analysis of drug company data that were submitted to the MHRA's safety review, reported that SSRIs did not appear to increase the risk of suicide attempts or thoughts [87].

Data regarding SSRIs and youth suicide are sparser, but no less controversial [12,13,88,89]. One study reported an inverse relationship between regional change in use of antidepressants and suicide [90]. Nonetheless, the highest possible standard should be applied to scientific data involving drug treatment of children because they are essentially involuntary patients: when a medication is prescribed for a young child, the adult caregiver ensures that the child takes the medication, regardless of the child's own desires. Yet, studies of adolescent compliance with medication treatment report notoriously low compliance outside of the controlled settings of clinical trials.

The sparse RCT findings suggest that improvement may not always be clinically significant. When evaluating a medication with side effects, potential clinical implications for later life, and questionable effectiveness, it should be compared to interventions such as exercise and CBT, which have shown some therapeutic effects on depression without medical side effects and risks [91]. Originally celebrated but recently disparaged by modern psychiatry, therapeutic rapport may prove more clinically significant than drug specificity.

## Conclusions

Given all of these limitations, patients and physicians should demand stronger evidence for the efficacy of antidepressants for CAD.

Some advocates assert that rather than using medication with side effects and low effectiveness, children should be offered interventions that produce therapeutic effects on depression without the medical side effects and associated risks [91]. However, clinicians and laypeople must apply comparable standards for evaluating the efficacy of drug and psychotherapy data. Whereas medical drug research occurs in a formally regulated, albeit imperfect, environment, safety and efficacy in psychotherapy research are largely unregulated. Moreover, unlike drug assays, psychotherapy studies do not typically report adverse events, their meta-analyses are sparse, and their experimental design lacks a placebo condition (see Table 2).

Finally, although antidepressants undoubtedly affect brain biochemistry, interpreting these neural changes is controversial, and a risk-benefit analysis of side effects and long-term health risks may cast a long shadow on the current preference for antidepressants as first-line treatment for CAD. Only more studies, and the passage of enough time to examine the putative long-term effects, will determine the efficacy of antidepressants in CAD. Clinicians, patients, families, and the public should be cognizant of these issues and exercise critical judgment as they make informed decisions.

## Abbreviations

CAD: child and adolescent depression
CBT: cognitive behavioral therapy
CI: confidence interval
FDA: Food and Drug Administration
MDD: major depressive disorder
MHRA: Medicines and Healthcare Products Regulatory Agency
RCT: randomized clinical trial
SSRI: selective serotonin reuptake inhibitor
TADS: Treatment for Adolescents with Depression Study
TCA: tricyclic antidepressant
